# CT-based application of the Iscan method on isolated ribs: reliability, accuracy, and the role of joint fossa sclerosis in age estimation on a contemporary European skeletal sample

**DOI:** 10.1007/s00414-026-03808-y

**Published:** 2026-05-02

**Authors:** Laura Filograna, Giulia Ceccobelli, Alessandro Mauro Tavone, Andrea Micillo, Raimondo Vella, Arianna D’Altorio, Flavia Chirico, Silvia Daria Beca, Alessandro Carini, Francesco Garaci, Maria Cristina Martinez-Labarga, Gian Luca Marella, Guglielmo Manenti

**Affiliations:** 1 Diagnostic Imaging and Interventional Radiology Policlinico Tor Vergata, Department of Biomedicine and Prevention, University of Rome Tor Vergata, Rome, 00133 Italy; 2 Department of Biomedicine and Prevention, Tor Vergata University of Rome, Via Montpellier 1, Rome, 00133 Italy; 3 Department of Surgical Sciences, Tor Vergata University of Rome, Via Montpellier 1, Rome, 00133 Italy; 4 Department of Biology, Tor Vergata University of Rome, Via della Ricerca Scientifica 1, Rome, 00133 Italy

**Keywords:** Post-mortem computed tomography, Human identification, Fourth rib, Age estimation, Forensic anthropology

## Abstract

This study evaluates the applicability of the Iscan method to Post-Mortem Computed Tomography (PMCT) for age estimation through morphological analysis of the sternal end of the fourth right rib. Employing a double-blind design, two observers independently assessed a sample of 112 fragments of fourth ribs from individuals of European origin, isolated and processed before CT imaging. The findings indicate that Iscan’s method applied via CT scans demonstrates strong reproducibility and substantial inter-operator reliability, as shown by weighted Cohen’s kappa values ranging from 0.75 (inter-operator) to 0.92–0.98 (intra-operator). The method exhibited higher accuracy and consistency for middle-aged and older individuals, particularly in phases 3 to 5, whereas younger and elderly age groups showed lower reliability, with phase 8 requiring further refinement due to significant variability. Furthermore, the study introduced and evaluated a new CT-specific parameter—joint fossa sclerosis—to enhance age-at-death predictions. Regression analysis incorporating this parameter demonstrated improved accuracy and refined age-range estimations, particularly highlighting sex-specific variations: in males, sclerosis tended to shift estimates toward higher age phases, while in females, it primarily improved precision within established phase limits without significantly altering phase assignments. These observations underscore potential sex-based differences in bone remodeling dynamics that influence the Iscan phase classification. This research paves the way for future identification, introduction, and refinement of additional morphological parameters aimed at enhancing predictive accuracy. The substantial age-range overlaps inherent to Iscan’s traditional method significantly limit its practical forensic applicability, emphasizing the need for methodological advancements to ensure reliable and accurate age estimation in forensic settings.

## Introduction

Age-at-death estimation in adults remains one of the most debated and methodologically complex issues in forensic anthropology. In real forensic contexts, age estimation contributes directly to personal identification, narrowing of missing persons lists, disaster victim identification, and judicial investigations. For forensic practitioners, the value of a method lies not only in statistical correlation with chronological age, but also in its reproducibility, transparency, and practical usefulness when applied to individual cases.

Several methods for estimating the age at death have been proposed, such as the analysis of the state of fusion of the cranial sutures, the analysis of symphyseal changes in the pubic bone [1, 2], and microscopic evaluation of intracortical remodelling in the long bones [3].

In the 1980s, Iscan and colleagues proposed a new method for assessing age at death based on the morphological study of the sternal end of the fourth right rib [4]. The choice of the fourth rib was purely conventional, being a bone fragment easily found during routine autopsy. This method involves the macroscopic examination of three structural components of the sternal end of the fourth rib, such as pit depth, pit shape, and rim and wall configurations. Initially, the method was applied to a sample of 118 white male subjects [5], and the fragments of the ribs belonging to this sample were divided into nine phases (from phase 0 to phase 8) corresponding to as many age ranges. Later, the same method was applied to a sample of fragments of the sternal end of the fourth right rib of 86 white women [6], for which nine new phases were created, taking into account the sexual dimorphism of the skeleton. Despite its historical importance and continued application, the Iscan method presents well-documented limitations: broad and overlapping age intervals, reliance on subjective morphological interpretation, and population-dependent variability. In practice, these features reduce its discriminative power, particularly in middle and advanced adult ages where phase overlap becomes substantial.

For this reason, in 2010, Hartnett [7] proposed a revision of the Iscan method by incorporating bone quality and density and reducing the number of phases (from phase 1 to phase 7). Although this revision narrowed certain age ranges, it did not fully resolve problems of overlap and has shown variable performance in older individuals. Thus, rib-based age estimation continues to face a structural limitation: the tension between reproducibility of phase assignment and the forensic utility of the resulting age intervals.

An innovative approach in anthropology known as “virtual anthropology” [8], based on computed tomography (CT) research, has been developed in recent years.

CT imaging offers a non-destructive, non-invasive and fast method of data collection and analysis, representing a perfect technique for visualizing bones not only with 2D reconstruction but with 3D volume rendering (VR) imaging, which flawlessly aligns with anthropological analysis [9, 10]. Moreover, it generates standardized digital records that can be repeatedly reviewed, measured and shared among observers, enhancing documentation consistency and reducing variability associated with direct macroscopic handling [8].

Our study aims to assess the reliability of CT imaging for age estimation based on the analysis of the sternal end of the right fourth rib from a contemporary European skeletal sample. The CT was used on dry, isolated bones. This approach serves two methodological purposes: first, to eliminate confounding variables such as soft tissue artifacts and positioning variability, thereby assessing the intrinsic reproducibility of CT-based phase evaluation and second, to explore whether CT visualization of internal bone characteristics provides additional age-related information not captured by traditional surface assessment.

Additionally, we aim to evaluate whether CT-specific parameters can refine age estimation beyond conventional phase assignment.

Therefore, the findings of this study could support forensic professionals by improving reproducibility, enabling standardized digital documentation, facilitating independent expert review, and potentially refining age estimates within overlapping phase ranges, thus improving methodological transparency and defensibility in casework.

## Materials and methods

### Study design, population, and specimens sampling and preparation

This study arises from a collaboration of the Institute of Legal Medicine and the Institute of Radiology of the University of Rome “Tor Vergata”, and was designed as an observational, double-blind reliability and agreement study of Iscan’s method for age estimation using CT reconstructions of the sternal end of the fourth right rib.

A convenience sample was used, comprising right fourth ribs collected at the Institute of Legal Medicine. The final dataset included 112 sternal rib ends from individuals of European origin with documented age at death. Cases were excluded in the presence of:

- anterior chest wall trauma,
- history or macroscopic evidence of chronic respiratory disease,
- congenital abnormalities or pathological alterations of the anterior chest wall.

Ethical approval was obtained from the Independent Ethics Committee of Policlinico Tor Vergata (protocol no. 71/17), authorizing bone retrieval during routine autopsies.

Ribs sampling followed a methodology already described in one of our previous morphological research in a wider sample [11]. During autopsy, rib segments were removed by incisions approximately 3 cm medially and laterally to the costochondral junction. Each specimen was labeled with a unique identification code to ensure pseudo-anonymization, while demographic data (gender, age, and height) were recorded separately.

Specimens underwent cleaning and preparation according to the described procedure [11] consisting of:

- maceration in soap and water to remove soft tissues,
- brief boiling (10–15 min) to facilitate debridement,
- manual removal of residual tissues and cartilage,
- air-drying for approximately 20 days.

### Radiological evaluation and data collection

After preparation, all fragments were scanned using using a 128-slice scanner (GE Medical System, Revolution CT) with the following parameters: slice acquisition 1.25 mm, pitch 0.5; rotation time 0.5 s, tube voltage 120 kVp, with adaptive mA. Images were reviewed and reconstructed using the MPR protocol, and 3D images were created using the Volume Rendering protocol for the CT evaluation.

**Fig. 1:**
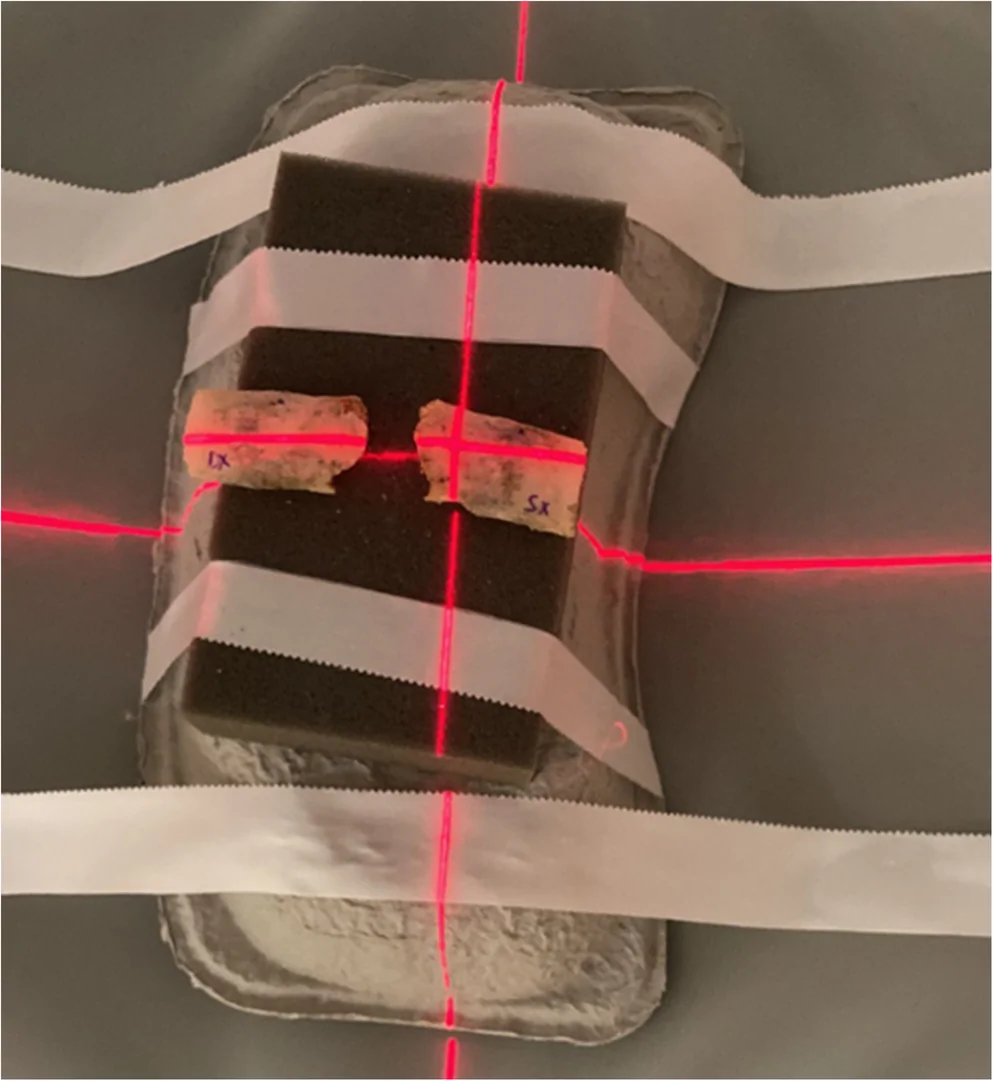
The image shows the placement of the bone specimen on the foam holder with associated laser CT-centering. The addition of the left bone specimen is to show the correct positioning following the anatomical orientation

Specimens were positioned anatomically on a radiolucent foam support to avoid reconstruction artifacts (Fig. 1).

Images were blindly analyzed by two radiologist observers in two different scoring sessions at a 6-month interval, to avoid memorization bias. Using MPR and VR reconstructions, each rib was classified according to Iscan’s phase system (phases 1–8), based on the CT-assessable morphological parameters:

1. progressive increase in pit depth,
2. transformation of pit shape (V-shaped to U-shaped),
3. changes in rim and wall morphology,
4. presence and development of osteophytes.

Table 1 summarizes the main characteristics used for classifying each phase and the corresponding age ranges for both sexes, according to Iscan [5, 6].

**Table 1:** -Iscan criteria for rib classification and phase-based age ranges

4th Rib Phase	Pit Depth	Pit Shape	Rim and Wall Morphology	Osteophytes	Iscan Phase-Based Age Males (y.o.)	Iscan Phase-Based Age Females (y.o.)
1	Shallow	Flat	Regular	-	17–18	-
2	Indented	V-shaped	Thick with rounded margins	-	18–25	16–20
3	Slightly shallow	Narrow U-shaped	Thick with smooth margins	-	19–33	20–24
4	Deep	U-shaped	Thin with irregular margins	-	22–35	24–40
5	Deep	U-shaped	Irregular, thin walls flaring outward	Small	28–52	29–77
6	Deep	U-shaped	Irregular, thin walls flaring outward	Moderate	32–71	32–79
7	Deep	U-shaped	Irregular, thin walls flaring outward	Long	44–85	48–83
8	Wide and deep	U-shaped	Irregular, thin walls flaring outward	Very long	44–85	62–90

**Fig. 2:**
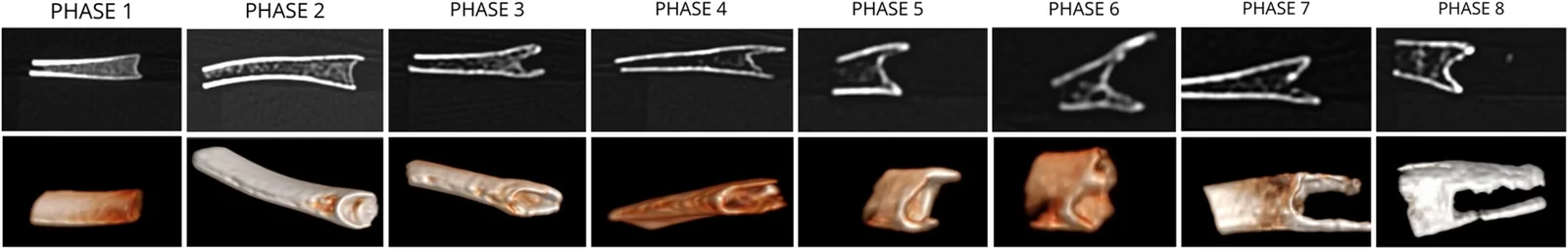
Top row: Axial CT images of bone specimens displayed in ascending Iscan phases (bone window). A progressive deepening of the joint fossa is clearly visible as the phase increases. Bottom row: 3D Volume Rendering reconstructions illustrating in detail the progressive appearance and elongation of osteophytes, which further characterize the later phases

Both raw pictures and multiplanar reconstructions were utilized to assess the depth and shape of the pit. To evaluate the rim and wall configuration visually, VR reconstructions were used. Each subject was placed in one of phases 1 through 8. Representative examples illustrating morphological features defining each Iscan phase are shown in Fig. 2.

During the assessment, it was observed that the samples also displayed variations in the degree of bone sclerosis. The samples were independently re-evaluated by the observers and assigned a sclerosis score on a three-level ordinal scale defined for this study:

- 0: no sclerosis,
- 1: sclerosis involving < 50% of the articular surface,
- 2: sclerosis involving > 50% of the articular surface.

This simple ordinal classification was designed to provide a reproducible visual assessment of sclerosis extent on CT images.

**Fig. 3:**
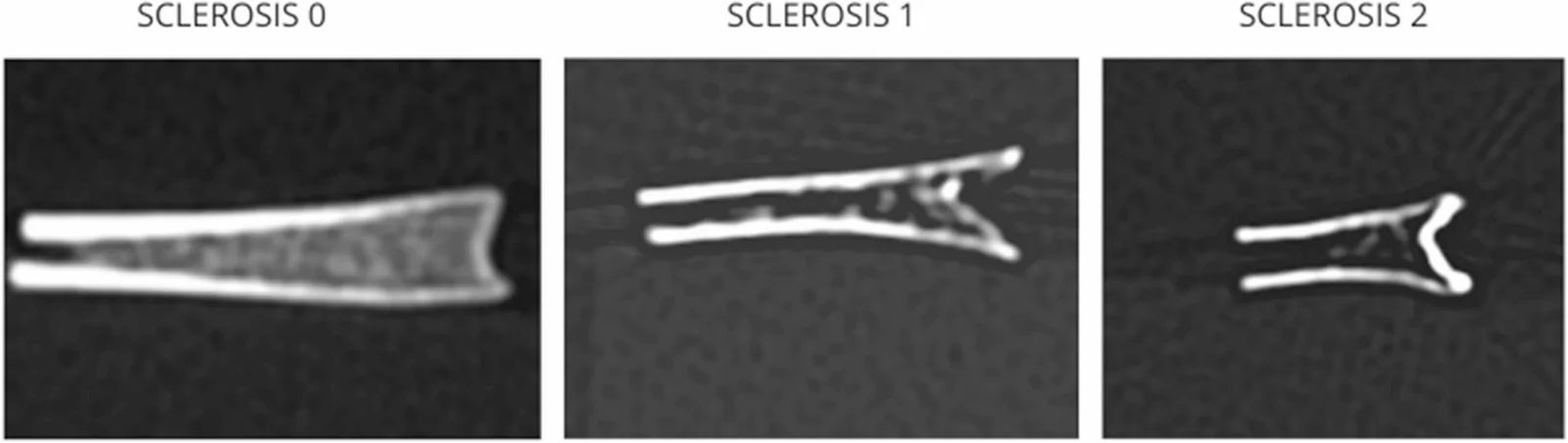
Visual representation of the sclerosis scoring system. The images illustrate the three degrees of sclerosis observed in the articular facet of the fourth right rib. Score 0: absence of sclerosis, with normal cortical bone appearance. Score 1: sclerosis affecting less than 50% of the joint surface, visible here as spots of cortical hyperdensities. Score 2: sclerosis affecting more than 50% of the joint surface, characterized by diffuse extent of cortical hyperdensity

Sclerosis was identified as cortical hyperdensity of the joint surface (Fig. 3). This parameter is easily quantifiable through direct observation and is highly reproducible across different evaluations. When necessary, common measurement tools can be used for a more precise estimation of sclerosis extent. Figure 3 provides a visual example of the three sclerosis score grades used in this study.

The measurements were conducted in a randomized sequence, with operators having no access to prior measurement data, clinical information, or any recorded details regarding the specimens. All data were collected using a simple numerical ID system for pseudo-anonymization, ensuring the specimens could be identified by gender while age details — stored in a separate database — remained inaccessible during the analytical phase.

### Statistics and reporting

A first descriptive analysis was performed. For each phase, the actual specimen age range was reported using means and standard deviation, as observed by each operator, separately for male and female subjects. To assess the expected observation for each operator, a prior probability was computed for each rib based on its documented age and the corresponding Iscan phase ranges, defined as:

$$\:{\mathrm{P}\mathrm{r}\mathrm{i}\mathrm{o}\mathrm{r}}_{\mathrm{p}\mathrm{h}\mathrm{a}\mathrm{s}\mathrm{e}}=\:\left\{\begin{array}{c}0\:\:,\:\:age\:\notin\:\:\left[{\mathrm{M}\mathrm{i}\mathrm{n}}_{\mathrm{p}\mathrm{h}\mathrm{a}\mathrm{s}\mathrm{e}},{\mathrm{M}\mathrm{a}\mathrm{x}}_{\mathrm{p}\mathrm{h}\mathrm{a}\mathrm{s}\mathrm{e}}\right]\\\:\:\\\:\:\\\:\frac{1}{\sum\:\mathrm{c}\mathrm{o}\mathrm{m}\mathrm{p}\mathrm{a}\mathrm{t}\mathrm{i}\mathrm{b}\mathrm{l}\mathrm{e}\:\mathrm{p}\mathrm{h}\mathrm{a}\mathrm{s}\mathrm{e}\mathrm{s}},\:\:age\:\in\:\left[{\mathrm{M}\mathrm{i}\mathrm{n}}_{\mathrm{p}\mathrm{h}\mathrm{a}\mathrm{s}\mathrm{e}},{\mathrm{M}\mathrm{a}\mathrm{x}}_{\mathrm{p}\mathrm{h}\mathrm{a}\mathrm{s}\mathrm{e}}\right]\:\:\end{array}\right.$$

For example, according to Iscan, a 30-year-old male may be correctly classified as phase 3, 4, or 5, giving each of those three phases a prior probability of 1/3, and zero for all others. Prior probability represents the theoretical phase distribution expected from chronological age used as a reference to evaluate operator classifications.

Inter- and intra-operator reliability were assessed using Weighted Cohen’s Kappa. In this analysis, agreement refers to exact phase assignment between observers and does not consider compatibility with the true age range, which was evaluated separately through the success metric.

For each observation, “success” was defined as assigning a phase compatible with the actual age at death; for each rib, the operator’s success rate was calculated as the number of successful classifications divided by the number of evaluations (two). Because the original Iscan phases present overlapping age ranges, different phase assignments may still be compatible with the same chronological age; therefore, this success metric reflects age compatibility rather than strict phase agreement. These success rates were plotted to obtain a curve modeled with a classical logistic function. To evaluate whether the two observers produced similar distributions of success rates across the examined ribs, differences between operators’ success rate distributions were assessed using the two-sample Kolmogorov–Smirnov test for equality of distributions, a non-parametric test that compares entire distributions without assuming normality, allowing detection of differences in the overall shape and dispersion of success rates rather than only differences in central tendency.

For each phase, prior and posterior probability densities were estimated using Kernel Density Estimation (KDE) with a Gaussian smoothing function and plotted. Kernel Density Estimation (KDE) is a non-parametric smoothing technique that provides a continuous and intuitive representation of probability distributions derived from discrete observations. It was chosen because it allows visualization of the overall pattern of the data without relying on arbitrary histogram binning, which may influence the apparent distribution of observations. In the present study, KDE was used to facilitate comparison between prior and posterior phase distributions and to highlight possible discrepancies between the expected and observed classification patterns.

Estimation error between observed and actual age (underestimation or overestimation) was analyzed by gender and age using Kernel Density Estimation (KDE) with a Gaussian smoothing function. This approach provides a smooth visualization of how estimation errors are distributed across age groups, making areas of higher concentration and possible systematic tendencies toward under- or overestimation easier to identify. The estimation error was represented continuously, with deviations from the centerline indicating the magnitude of the error: larger deviations correspond to greater misclassification. Individual observations were overlaid on the KDE to provide direct visual insight into the distribution of errors across different age groups and sexes.

The Guidelines for Reporting Reliability and Agreement Studies (GRRAS) were used for reporting the results, and statistical significance was set at *P* = 0.05 for all inferential analyses [12].

To incorporate the sclerosis score and potentially narrow the broad age ranges associated with each phase, two linear regression models were fitted, with the subject’s actual age as the dependent variable. The first model includes only the CT Iscan phase (treated as an ordinal variable), and the second model includes both the Iscan phase and the sclerosis score as predictors. Model performance was compared using changes in the coefficient of determination (R²) and mean squared error (MSE). For each phase and sclerosis category, 95% confidence intervals for the estimated ages were computed to determine whether including sclerosis yielded narrower intervals. A significant improvement in R², a reduction in MSE, or narrower confidence intervals in the extended model was considered evidence that the sclerosis score refines age estimation within each phase. The comparison between the two models was intended to evaluate whether the inclusion of the sclerosis score provided additional predictive information beyond the phase classification alone.

## Results

### Descriptive analysis

The collected sample consisted of 112 fourth right ribs, collected during the autopsies performed at the Institute of Legal Medicine of the University of Rome “Tor Vergata,” between July 2017 and September 2023. The sample consisted of 74 male and 38 female subjects. The mean age was 53.4 years (SD: 14.1) for males, and 58.0 (SD: 23.8) for females. The sample distribution, by phase, is detailed in Table 2. Several ribs could correspond to many phases, as Iscan’s age ranges are notoriously wide and overlapping. Two observers (L. F. and A. M.) observed the 112 ribs twice each, for a total of 448 observations. For each phase, the mean and standard deviation are reported in Table 2.

**Table 2:** -Mean and SD of actual rib age for each observed phase, by gender, classification type and operator

Gender	Phase	*N* of ribs (compatible within phase)	Expected *n* by each operator	Observed *n* by OP 1	OP 1 mean	OP 1 SD	Observed *n* by OP 2	OP 2 mean	OP 2 SD
Female	1	0	0	3	17.00	-	6	19.67	4.62
	2	2	4	9	22.00	3.32	8	24.50	4.04
	3	2	4	10	45.60	18.80	6	50.00	23.52
	4	6	7	16	53.50	17.72	6	60.00	20.41
	5	22	15	4	59.00	1.41	21	62.58	21.30
	6	21	14	6	62.33	13.32	13	76.29	8.2
	7	22	15	19	76.90	9.75	8	69.80	8.47
	8	17	16	9	85.80	4.66	8	77.60	16.88
Male	1	1	1	2	18.00	-	2	18.00	-
	2	1	1	19	38.40	12.53	12	38.00	8.06
	3	7	5	24	43.92	12.49	18	39.36	10.72
	4	9	6	20	55.20	6.83	27	52.93	8.55
	5	30	20	25	53.62	10.62	31	56.00	13.24
	6	60	40	26	60.38	11.75	26	58.73	11.44
	7	57	38	26	60.00	7.97	22	59.92	10.09
	8	57	38	6	77.33	4.62	10	63.83	11.99

### Agreement and reliability

Intra-operator agreement was found to be almost perfect for both operators, with weighted Cohen’s kappa values of 0.98 (Op.1) and 0.92 (Op.2); inter-operator agreement was substantial, both in the first (kappa = 0.75) and in the second (kappa = 0.76) session of scoring. The *P*-values were < 0.001 for all estimations.

**Fig. 4:**
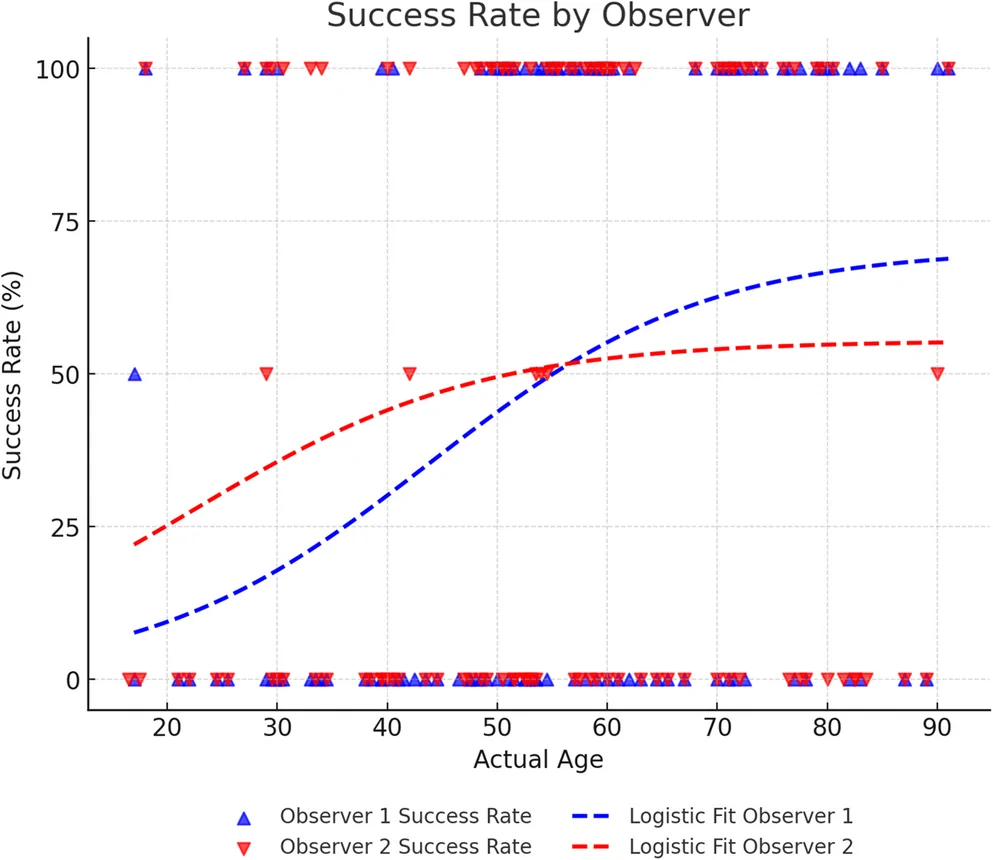
Success rate by operator. Scatter points represent the success rates for each observed case, with Observer 1 shown as upward triangles and Observer 2 as downward triangles. Dashed lines represent logistic regression fits for each observer

The success rate for phase assignment was found to be 46.9% for Observer 1 and 48.2% for Observer 2. The Kolmogorov-Smirnov test showed no significant difference between the distributions of success rates (KS = 0.036, *P* = 0.999), indicating that the two observers performed similarly in assigning rib phases. Overall success rates are depicted in Fig. 4.

**Fig. 5:**
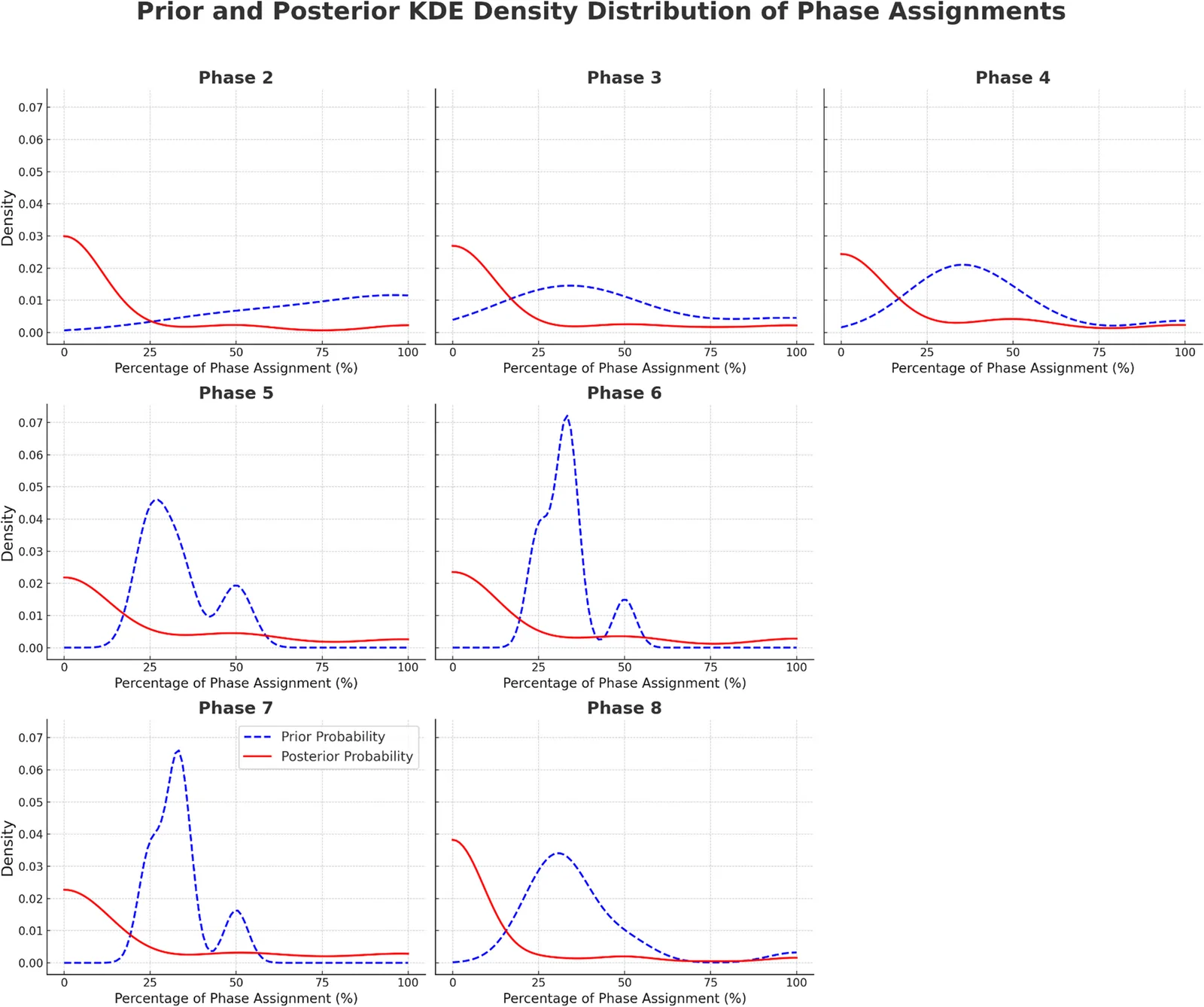
Prior and Posterior KDE Density Distribution of Phase Assignments: This figure compares the expected (Prior, blue dashed line) and observed (Posterior, red solid line) phase assignments using Kernel Density Estimation (KDE) for phases 2 to 8. The Prior distribution reflects the theoretical classification based on the Iscan method, while the Posterior distribution represents actual phase assignments made by two independent observers. The degree of misalignment between the two curves highlights potential biases in phase estimation: Rightward shifts in the red curve indicate overestimation of that phase. Leftward shifts suggest underestimation. Phase 1 was omitted as no observation had a prior probability of being phase 1

Figure 5 shows the Prior and Posterior KDE Density Distributions for the Iscan CT classification. Prior probability is overrepresented in phases 6 and higher, with Posterior distributions shifted rightward, indicating a tendency toward higher-phase assignments. Differences are minimal in phases 3 to 5, where Prior and Posterior distributions align more closely. The largest discrepancy occurs in phase 8, where the Posterior density is more dispersed, suggesting greater classification variability.

**Fig. 6:**
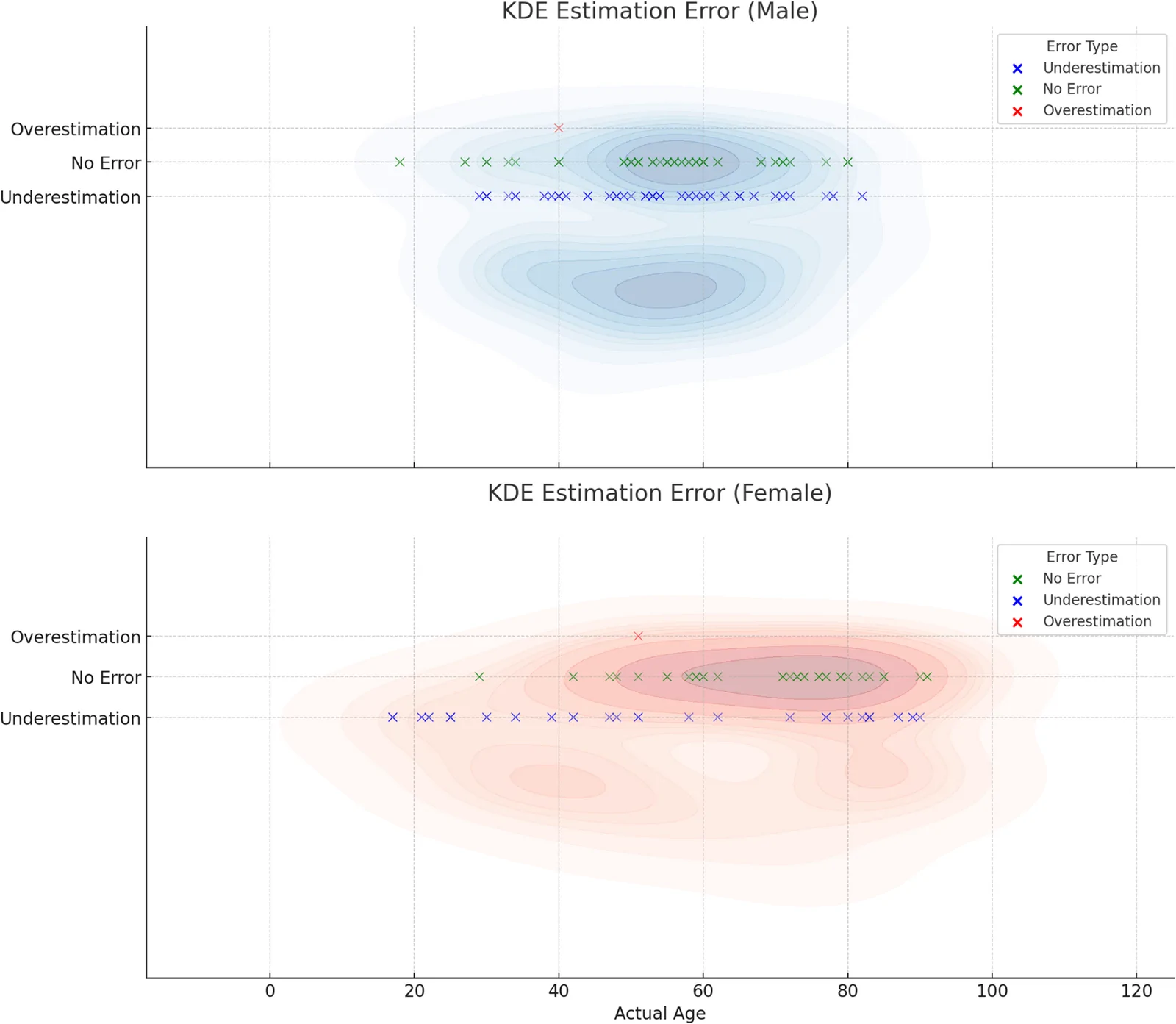
Kernel Density Estimation (KDE) plots illustrating estimation errors for males (top) and females (bottom). The X-axis represents the actual age of individuals, while the Y-axis reflects the estimation error. Each point corresponds to a single observation, with colors indicating the type of error: blue for underestimation, green for correct classification, and red for overestimation. In the background, the KDE heatmap represents the density of observations. Darker regions indicate a higher concentration of cases, helping to visualize where errors are more frequent. The color scheme (blue for males and red for females) is purely stylistic and does not indicate error magnitude. The position of denser regions along the Y-axis provides insight into error trends: Regions shifting upward indicate grade and tendency toward overestimation; Regions shifting downward suggest grade and tendency toward underestimation; The closer a region is to the center, the more accurate the classification

To highlight differences in accuracy, we investigated the tendency to under- or over-estimate, by gender, according to the specimen age. A depiction of each method’s misclassification tendency, distinct by gender, is available in Fig. 6. The method showed a tendency to underestimate both sexes.

### Relation between score and age, and implementation of sclerosis score

To evaluate the relationship between age and observation on the ribs, for each sex, two linear regression models were tested. The first model was based solely on the CT Iscan phase classification, treating it as an ordinal variable. The second model incorporated both the CT Iscan phase and the degree of sclerosis as independent variables, aiming to assess whether the latter could refine age estimation. Each observation was considered individually.

#### Males

The tests were based on a total of 296 observations (74 male individuals × 4 observations each). The first model, using only the CT Iscan phase, explained 39% of age variability (R² = 0.39) with a mean squared error (MSE = 119.28 years²). The second model, incorporating the sclerosis score, improved explanatory power (R² = 0.49) and reduced prediction error (MSE = 99.42 years²). However, the 95% confidence interval width increased from 3.43 to 3.78 years, indicating slightly greater variability in individual predictions. The analysis of residuals with the Shapiro-Wilk test indicated slight deviations from normality (W = 0.98). Given the W statistic values very close to 1 the observed deviations are minimal and practically negligible, especially considering the relatively large sample size. Therefore, the assumptions underlying the linear regression model can be regarded as sufficiently satisfied for the purposes of this study.

**Fig. 7:**
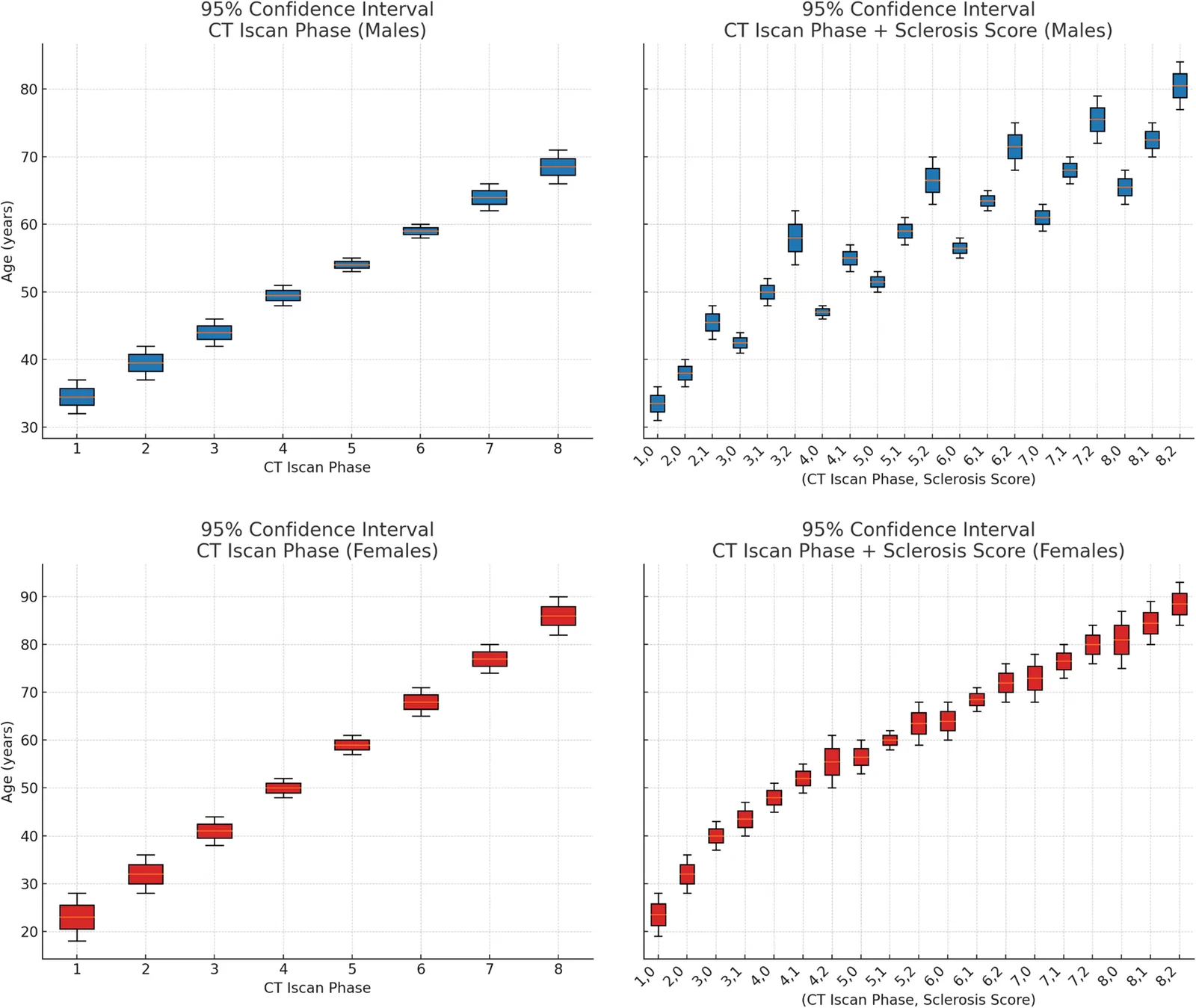
Boxplots representing the 95% confidence intervals (CI) for age estimation. The left plot shows the CI for the model 1, which estimates age using only CT-derived phase classification. The right plot displays the CI for the model 2, which integrates sclerosis scoring to refine age estimation. Each box represents the range between the lower and upper CI bounds, with whiskers extending to the full interval

Table 3 shows the predicted 95% confidence intervals for age estimation in both models and sexes, highlighting the effect of integrating the sclerosis score. The confidence intervals for the CT Iscan Phase model are generally broader, while the CT Iscan Phase + Sclerosis Score model refines the estimates by reducing the interval width for most phases. Figure 7 visually represents these confidence intervals using box plots, illustrating the variability in estimated age ranges across phases and sclerosis scores. As the overall width increases slightly from 3.43 to 3.78 years, suggesting that individual-level variability increases in some phases, this effect is not uniform. In the middle phases, where the model has the highest predictive accuracy, sclerosis refines age estimates. For instance, in Phase 5, the confidence interval shifts from 53 to 55 years in the CT Iscan Phase model to 50–53 years for a sclerosis score of 0 and 57–61 years for a sclerosis score of 1, indicating a more precise classification. In contrast, for older individuals (Phases 6–8), the confidence intervals widen, as seen in Phase 6, where the range extends from 55 to 58 years (sclerosis score 0) to 68–75 years (sclerosis score 2).

**Table 3:** Comparison between estimated confidence intervals for the two models in both sexes

Gender	MODEL1: CT Iscan Phase only	CI Lower bound	CI Upper bound	MODEL2: CT Iscan phase + sclerosis score	CI Lower bound	CI Upper Bound
Male	1	32	37	1 − 0	31	36
	2	37	42	2 − 0 2 − 1	36 43	40 48
	3	42	46	3 − 0 3 − 1 3 − 2	41 48 54	44 52 62
	4	48	51	4 − 0 4 − 1	46 53	48 57
	5	53	55	5 − 0 5 − 1 5 − 2	50 57 63	53 61 70
	6	58	60	6 − 0 6 − 1 6 − 2	55 62 68	58 65 75
	7	62	66	7 − 0 7 − 1 7 − 2	59 66 72	63 70 79
	8	66	71	8 − 0 8 − 1 8 − 2	63 70 77	68 75 84
Female	1	18	28	1 − 0	19	28
	2	28	36	2 − 0	28	36
	3	38	44	3 − 0 3 − 1	37 40	43 47
	4	48	52	4 − 0 4 − 1 4 − 2	45 49 50	51 55 61
	5	57	61	5 − 0 5 − 1 5 − 2	53 59 60	60 62 68
	6	65	71	6 − 0 6 − 1 6 − 2	60 66 68	68 71 76
	7	74	80	7 − 0 7 − 1 7 − 2	68 73 76	78 80 84
	8	82	90	8 − 0 8 − 1 8 − 2	75 80 84	87 89 93

*For model 2 the first number indicates the phase and the second indicates the sclerosis score (e.g. 4 − 1 stands for phase 4 - sclerosis score 1)*

Additionally, the inclusion of sclerosis modifies the relative positioning of age estimates across phases, leading to overlapping and exceeding effects. In Phase 6, individuals with a sclerosis score of 2 have an estimated age range of 68–75 years, surpassing the 62–66 years observed in Phase 7 with a sclerosis score of 0. Similarly, in Phase 3, the range for a sclerosis score of 2 (54–62 years) exceeds that of Phase 4 with sclerosis 0 (46–48 years). The overlapping and exceeding effects indicate that sclerosis modifies age distribution within phases, shifting estimated ranges beyond those of the subsequent phase with sclerosis 0.

#### Females

The tests were based on a total of 152 observations (38 female individuals × 4 observations each). The first model, using only the CT Iscan phase, explained 64% of age variability (R² = 0.64) with a mean squared error (MSE = 200.78 years²). The second model, incorporating the sclerosis score, minimally improved explanatory power (R² = 0.65) and reduced prediction error (MSE = 195.51 years²). However, the 95% confidence interval width increased from 6.25 to 7.60 years, indicating slightly greater variability in individual predictions.

The analysis of residuals with the Shapiro-Wilk test indicated slight deviations from normality (W = 0.95). Given the W statistic values close to 1, the observed deviations are minimal and practically negligible, especially considering the relatively large sample size. Therefore, the assumptions underlying the linear regression model can be regarded as sufficiently satisfied for the purposes of this study.

Table 3 presents the 95% confidence intervals for age estimation in both models, illustrating, for females, the effect of integrating the sclerosis score. Compared to males, the impact of sclerosis follows a more linear pattern, with fewer overlapping and exceeding effects. Figure 7 visually represents these confidence intervals using box plots, showing the distribution of estimated age ranges across phases and sclerosis scores.

Unlike the male sample, the effect of widening of confidence interval in the second model is more evenly distributed across phases, without strong deviations in specific age groups. While the overall trend of age estimation remains progressive and structured, the introduction of sclerosis still leads to moderate adjustments in estimated age ranges within phases. However, these adjustments do not significantly disrupt the sequential progression of CT Iscan phases. The pattern observed in males, where individuals with high sclerosis in a lower phase could exceed the estimated range of the next phase, is less prominent in females. Instead, sclerosis appears to act as a gradual modifier, refining age estimates without altering the overall phase-based progression.

## Discussion

A study has been conducted on the applicability of the Iscan method for assessing age at death via morphological analysis of the sternal end of the fourth right rib.

CT imaging is a crucial method for visualizing bone structure and its internal and external architecture in a non-invasive, non-destructive, and extremely rapid manner.

To our knowledge, this is the first study in literature applying the Iscan method to CT scans performed directly on isolated and treated rib specimens. Previous studies have demonstrated the applicability of the Iscan method using CT scans; however, they analyzed images obtained from post-mortem whole-body CT scans [13], living subjects [10], or clinical databases [14], indirectly reconstructing images of the costochondral joint. Our approach differs, as we physically sampled the rib specimens during autopsy—an approach previously described only by Dedouit et al. [8], who did not further process the specimens—then cleaned and prepared them before CT scanning. Although more complex, this methodological choice allowed us to optimize the visualization of morphological features, minimize potential positioning artifacts, and work under controlled and standardized conditions.

Initially, in our study, a descriptive analysis was carried out on the sample consisting of 112 fragments of the fourth rib, of which 74 came from male subjects and 38 came from female subjects. Then, the 112 ribs were scanned by CT, and the images obtained were analyzed and classified according to the Iscan method by two observers. Each observer made two observations of the 112 fragments of the fourth rib.

An agreement and reliability study was subsequently conducted between the two observers.

It was observed that the intra-observer agreement between the first and second scoring sessions is almost perfect: for the first observer, the value of the K-weighted is 0.980 (p-value < 0.01); for the second observer, the value of the K-weighted is 0.923 (p-value < 0.01).

The agreement between the evaluators’ judgements in the first and second scoring sessions is substantial: for the first scoring session, the concordance between the observers’ judgements has a K-weighted value of 0.749 (p-value < 0.01); for the second scoring session, the concordance between the observers’ judgements has a K-weighted value of 0.759 (p-value < 0.01).

Although the statistical measures are not directly comparable, similar results were observed by Dedouit et al. [8], who applied the Iscan method to PMCT for the assessment of age at death and obtained a substantial intra-operator reliability confirmed by a Krippendorff’s alpha coefficient of 0.79 as well as excellent inter-operator reliability confirmed by a Krippendorff’s alpha coefficient between 0.78 and 0.86.

Blaszkowska et al. [14] obtained for intra-operator concordance a substantial K-weighted value of 0.76 and an excellent intra-observer concordance with a K-weighted value of 0.825.

Recently, Beltran-Aroca et al. [15] have obtained results similar to those of our study, observing an almost perfect intra-operator agreement with a K-weighted value of 0.954 for CT images and a substantial inter-operator agreement with a K-weighted value of 0.727, again for CT scans.

When analyzing success rates by age groups in the present study, the highest accuracy was observed in the 76 and over age group for Observer 1 and the 46–60 age group for Observer 2, where phase assignments were more consistent with actual age. Conversely, the lowest success rates were found in the 31–45 age group for both observers, where misclassifications were more frequent. These findings suggest that Iscan’s method is more reliable for middle-aged and older individuals but shows limitations in both younger and elderly individuals.

It should be noted that phase agreement and classification success represent two different aspects of the method’s performance. In the present study, agreement refers to strict concordance in phase assignment between observers, whereas success reflects whether the assigned phase is compatible with the individual’s chronological age according to the original Iscan age ranges. Because these age ranges are intentionally broad and overlapping, different phase assignments may still correspond to the same age interval. Consequently, lower phase agreement may coexist with relatively high age-compatible success rates, reflecting an intrinsic characteristic of the Iscan phase system rather than a methodological inconsistency.

In terms of Iscan phases, phase 7 exhibited the highest success rate for Observer 1, while phase 6 had the highest accuracy for Observer 2, aligning well with expected age ranges. These results are partly in agreement with those obtained by Beltran-Aroca et al. [15], whose estimates were more accurate in stages 6 and 8. On the other hand, phase 3 had the lowest success rate for Observer 2, often leading to misclassifications. Finally, the low success rate for phase 1/Observer 1 is not meaningful for evaluating the method’s accuracy, since phase 1 did not include any cases with a corresponding actual ag.

This confirms that the method struggles to classify younger individuals accurately, while it performs more reliably in later phases.

The observed differences between Prior and Posterior KDE Density Distributions highlight systematic variations in the classification of rib phases using the Iscan method applied to CT imaging. The overrepresentation of higher phases in the Prior probability suggests that the Iscan model inherently expects a broader distribution of individuals in later phases. However, the rightward shift in Posterior distributions for phases 6 and above indicates a tendency for observers to assign higher phases more frequently than expected, potentially leading to overestimation of age at death in older individuals. The alignment between Prior and Posterior distributions in phases 3 to 5 suggests that these phases are more stable and reliably assigned.

These results contrast with those observed by Beltran-Aroca et al. [15] whose study shows a tendency to overestimate age in the early stages (1–3) and to underestimate age in the later stages (4–8), with two exceptions from phase 3, which tends to be underestimated, and phase 7, which tends to be overestimated.

The present study also shows an increased dispersion in phase 8, indicating greater variability in observer classifications. This finding likely reflects the nature of skeletal remodeling in advanced ages. In later stages of rib metamorphosis, morphological changes tend to become less clearly distinguishable, and the progression of degenerative features such as rim irregularity, osteophyte development, and cortical remodeling may occur in a more heterogeneous and individualized manner. As a consequence, specimens belonging to older age groups may present combinations of morphological features that do not perfectly correspond to a single phase description, increasing the subjective component of classification.

When evaluated through CT imaging, this variability may become even more apparent. Unlike traditional macroscopic examination, CT visualization allows the observer to appreciate both external morphology and internal structural alterations, including cortical density changes and joint surface sclerosis. These additional details may reveal differences between specimens that would otherwise be grouped within the same phase when using purely surface-based criteria.

In this context, the broad morphological spectrum currently included within phase 8 may encompass individuals at different stages of advanced skeletal remodeling. The dispersion observed in our results therefore suggests that this phase may represent a heterogeneous category rather than a single morphological stage. Future CT-based applications of the method could benefit from exploring a possible subdivision or refinement of the criteria defining this phase, in order to better capture the variability observed in older individuals and potentially improve classification consistency in advanced age groups.

Overall, the results emphasize the need to consider potential biases in phase-based age estimation methods, especially for older individuals, where classification variability increases.

Indeed, as previously stated by Russell et al. [16], one of the main limitations of the Iscan and Hartnett methods is that they are not accurate in describing the weight and quality of the bone, which are two decisive parameters in assigning a sample to the correct age stage, especially for elderly individuals. Furthermore, these are two bone characteristics that can only be assessed by touch, so this could be a limit, even in the case of applying the Iscan method to PMCT [15].

In 2018, Merritt [17] proposed a revised method for applying the Iscan and Hartnett methods to CT scans of the fourth rib that also considered weight and bone quality; through this new method an increase in the accuracy of assigning specimens to the correct age phase was achieved, especially for male subjects in the 30 years range and female subjects under 40 years of age.

Further investigation could explore whether integrating additional morphological parameters or revising phase definitions could improve the reliability of the Iscan method in forensic and anthropological contexts. Relative to this, our study shows how, by introducing a new CT-specific parameter (joint fossa sclerosis), additional information about the age of the individual from the cadavers can be obtained, further narrowing the age ranges.

As previously stated, three patterns of joint fossa sclerosis were identified by PMCT: a score of 0 was assigned if joint fossa sclerosis was not present; a score of 1 was assigned if the sclerosis involved < 50% of the joint surface; a score of 2 was assigned if the sclerosis involved > 50% of the joint surface. Some age groups (e.g., groups 1 and 2) fall solely into the sclerosis group 0, while for other age groups, the presence of sclerosis scores of 1 or 2 may lean toward higher ranges for corpse age-prediction. To our knowledge, no previous study on the application of the Iscan method to PMCT for the estimation of age at death has ever shown the presence of sclerosis of the fossa of the sternal end of the fourth rib.

The regression models from male subjects suggest that while the sclerosis parameter enhances overall age estimation accuracy, it introduces a minor increase in uncertainty for single-case assessments. These findings suggest that sclerosis is a structural modifier of phase-based classification rather than a simple additive factor. While it enhances differentiation among individuals within the same phase, it introduces greater variability in some age groups, particularly in older individuals, where sclerosis progresses more irregularly. The redistribution of estimated ages challenges the sequential interpretation of CT Iscan phases, indicating that skeletal aging follows a more individualized trajectory, with sclerosis playing a key role in accelerating or modifying expected age estimations. It has been observed that in male subjects, the presence of sclerosis leads to assigning the individual to a higher age phase. For example, an individual in stage 2 with a sclerosis score of 2 will be more likely to be assigned to a higher stage, compared to an individual in stage 3 with a sclerosis score of 0.

Conversely, findings of the regression models from female subjects suggest that while sclerosis increases individual-level variability, it does not create major inconsistencies in phase-based classification for females. Instead, it follows a more structured and predictable effect, modifying estimated ages within expected limits rather than shifting individuals into the next phase’s range. This pattern reinforces the importance of integrating sclerosis into forensic age estimation models but also highlights potential sex-based differences in how bone remodeling and sclerosis progression interact with phase-based classification.

It is therefore crucial to highlight how this new parameter should not be used alone in assigning samples to the correct age stage but as an additional evaluation to the Iscan method applied to PMCT.

From a practical forensic perspective, CT-based evaluation of the sternal end of the fourth rib may have several applications in identification contexts. In many forensic scenarios, particularly in cases of advanced decomposition, skeletonized remains, or mass disaster investigations, isolated rib fragments may be among the preserved skeletal elements available for analysis. The possibility of applying the Iscan method through CT imaging allows non-destructive examination, digital archiving of morphological features, and repeated evaluation by multiple experts, which may improve transparency and reproducibility in forensic casework.

In addition, the increasing use of post-mortem computed tomography (PMCT) in forensic practice makes CT-based rib analysis potentially applicable even before skeletal preparation, allowing preliminary age estimation during radiological examinations.

Although the Iscan method was originally developed for age-at-death estimation, CT-based evaluation of rib morphology could also represent a complementary indicator in radiological age estimation of living individuals. In such contexts, non-invasive imaging is essential, and internal bone features such as sclerosis may provide additional information not accessible through traditional examination. However, further validation on clinical datasets would be necessary before considering such applications in medico-legal practice.

Further studies are needed to improve this method of applying the Iscan method to CT scans of the sternal end of the fourth rib to determine the age at death from skeletal remains and also to determine its potential application in determining the age of living subjects for legal purposes [18].

## Limits

This study has some limitations. The study population was not equally represented between males and females, with a higher number of male specimens included in the dataset. This imbalance may influence the robustness of sex-specific analyses in several ways. First, the smaller female subsample reduces statistical power and increases the variability of regression estimates, potentially contributing to the wider confidence intervals observed in the female models. Second, unequal group sizes may amplify the influence of individual observations in the smaller group, making the regression results more sensitive to outliers or local distribution patterns. Consequently, the differences observed between male and female regression models should be interpreted cautiously and considered exploratory rather than definitive evidence of sex-related differences in rib aging patterns. In addition, the unequal distribution may partially affect the apparent role of the sclerosis parameter between sexes, since smaller samples can obscure or exaggerate structural relationships between variables. Future studies including larger and more balanced sex distributions will be necessary to determine whether the observed differences reflect true biological variation in skeletal remodeling or are partly attributable to sampling.

## Data Availability

The datasets generated during and analysed during the current study are available from the corresponding author on reasonable request.
